# RNA-clique: a method for computing genetic distances from RNA-seq data

**DOI:** 10.1186/s12859-024-05811-9

**Published:** 2024-06-04

**Authors:** Andrew C. Tapia, Jerzy W. Jaromczyk, Neil Moore, Christopher L. Schardl

**Affiliations:** 1 Department of Computer Science, University of Kentucky, 329 Rose St, Lexington, KY 40508 USA; 2 Department of Plant Pathology, University of Kentucky, 1405 Veterans Dr, Lexington, KY 40546 USA

**Keywords:** RNA-seq, Genetic distance, Graph algorithms, Phylogenetics

## Abstract

**Background:**

Although RNA-seq data are traditionally used for quantifying gene expression levels, the same data could be useful in an integrated approach to compute genetic distances as well. Challenges to using mRNA sequences for computing genetic distances include the relatively high conservation of coding sequences and the presence of paralogous and, in some species, homeologous genes.

**Results:**

We developed a new computational method, RNA-clique, for calculating genetic distances using assembled RNA-seq data and assessed the efficacy of the method using biological and simulated data. The method employs reciprocal BLASTn followed by graph-based filtering to ensure that only orthologous genes are compared. Each vertex in the graph constructed for filtering represents a gene in a specific sample under comparison, and an edge connects a pair of vertices if the genes they represent are best matches for each other in their respective samples. The distance computation is a function of the BLAST alignment statistics and the constructed graph and incorporates only those genes that are present in some complete connected component of this graph. As a biological testbed we used RNA-seq data of tall fescue (*Lolium arundinaceum*), an allohexaploid plant ($2n = 14\text { Gb}$), and bluehead wrasse (*Thalassoma bifasciatum*), a teleost fish. RNA-clique reliably distinguished individual tall fescue plants by genotype and distinguished bluehead wrasse RNA-seq samples by individual. In tests with simulated RNA-seq data, the ground truth phylogeny was accurately recovered from the computed distances. Moreover, tests of the algorithm parameters indicated that, even with stringent filtering for orthologs, sufficient sequence data were retained for the distance computations. Although comparisons with an alternative method revealed that RNA-clique has relatively high time and memory requirements, the comparisons also showed that RNA-clique’s results were at least as reliable as the alternative’s for tall fescue data and were much more reliable for the bluehead wrasse data.

**Conclusion:**

Results of this work indicate that RNA-clique works well as a way of deriving genetic distances from RNA-seq data, thus providing a methodological integration of functional and genetic diversity studies.

**Supplementary Information:**

The online version contains supplementary material available at 10.1186/s12859-024-05811-9.

## Background

In this paper, we describe and evaluate RNA-clique, a new approach for computing genetic distance matrices using only RNA-seq data. The method employs rigorous filtering for alignments of orthologous transcripts and uses as its input sets of RNA-seq samples from individuals being compared. The computed distance is a function of alignment statistics and a graph representing inferred orthologies between genes in the set of samples.

This work is key to an NSF-funded project in the Dimensions of Biodiversity program by providing a novel approach to integrate studies of functional diversity (in this case, RNA-seq) and genetic diversity. The technique is to be applied to plant population surveys to assess the interaction of plant genetic diversity to response to environmental variables and diverse symbiotic microbes. Typically, genetic distances are computed using whole, or, more often, partial genomic DNA sequences. Genomic DNA sequences are well-suited for such calculations—they allow us to detect precisely the differences in the genome sequences of two or more individuals. Unfortunately, obtaining genomic DNA sequences can also be costly, especially for organisms with large genomes such as vertebrates or vascular plants.

RNA-seq data are typically used for identifying and measuring expression levels of genes, and RNA-seq studies compare gene expression among multiple individuals or the same individual under different conditions. Since transcripts mostly reflect genomic DNA (aside from splicing and, rarely, RNA-editing), there is potential for using RNA-seq for computing genetic distances as well. A way of computing genetic distances using RNA-seq data would be convenient and economical for projects that need RNA-seq data for other purposes but do not need genomic DNA sequences for any other applications.

**Fig. 1:**
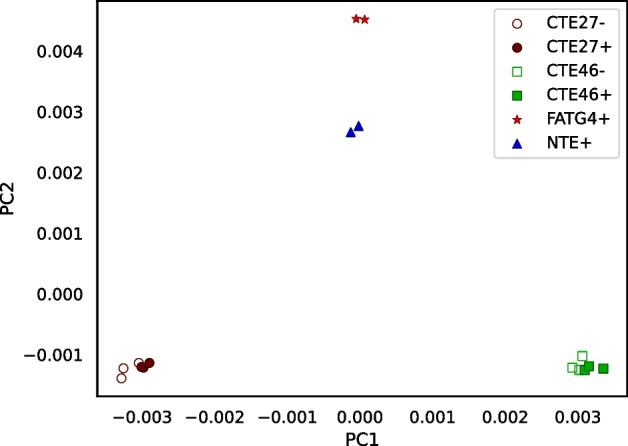
PCoA plot for the distance matrix computed for a set of 16 RNA-seq samples. Each sample represents a clone of one of four genotypes of the grass tall fescue (*Lolium arundinaceum*). Genotypes are designated CTE27, CTE46, FATG4, and NTE. Presence ($+$) or absence (−) of endophyte (the symbiotic fungus *Epichloë coenophiala*) was relevant to the original gene expression studies [2]

The method we propose takes a cautious approach by stringently filtering the sequences used for estimating distances. Thus, the way we use RNA-seq data is analogous to a reduced-representation genome sequencing [1]. Because we filter so much data and because most transcribed sequence is coding sequence, which is more highly conserved than other regions of the genome, a potential problem is retaining sufficient variation to discriminate between individuals. Hence, we test RNA-clique with multiple RNA-seq samples from each of four plants derived from one ecotype. The results indicated the feasibility of the approach described (Fig. 1).

Existing tools for computing genetic distances using RNA-seq data alone are scarce. One possible option is the approach implemented in the Cnidaria software of Aflitos et al. [3]. Cnidaria can operate on either raw RNA-seq reads or assembled transcriptomes. The software uses a “*k*-mer counting” approach. The simplest variation of the approach implemented in Cnidaria computes the distance between two samples as the Jaccard distance between the intersections of the sets of *k*-mers that appear in the sequences of the two samples with those that appear in at least two samples. (The Jaccard distance is taken to be 1 minus the Jaccard similarity. The Jaccard similarity is the number of elements in the sets’ intersection divided by the number of elements in the sets’ union. Since the similarity is a ratio of counts of elements, *k*-mers, in this case, both the similarity and distance are dimensionless.)

Cnidaria computes distances without alignment—the input sequences are neither aligned to a reference genome nor to each other. The *k*-mer counting approach instead works on the principle that similar sequences share more *k*-mers. This means that orthologous sequences are not directly identified and compared, and we are concerned that results might be influenced by paralogous genes or, in the case of polyploid organisms, sets of homeologs. In this paper, we propose an approach in which orthologous sequences from RNA-seq data are identified and compared directly. We also compare RNA-clique with Cnidaria in terms of accuracy of results and resource usage (“Distance tests and Resource usage tests” sections).

RNA-clique utilizes a graph to represent orthology relationships among genes in the samples considered. The graph produced as part of our method bears some resemblance to those built for finding the Clusters of Orthologous Groups (COGs) of Tatusov et al. [4]. The graph of RNA-clique differs from that of COG in that the edges represent a bidirectional best match between genes (or a non-empty intersection between the top *N* best matches in both directions if the parameter $N > 1$), whereas the edges in the COG graph may represent a unidirectional best match between proteins. Additionally, the eponymous subgraphs identified by the COG method consist of proteins inferred to be related as either orthologs or paralogs. In contrast, the “ideal” components of our method (described in “Computing distances for multiple samples” section) contain genes inferred to be related only as orthologs. COG does also identify some subgraphs presumed to be related as orthologs only—triangles (cliques with exactly three vertices) are “minimal COGs” in which each pair of proteins is orthologous. The ideal components of our method may be viewed as an of extension of this idea, since every ideal component is a clique. Furthermore, every ideal component is a COG (ignoring the distinction between genes and proteins), but not vice versa.

Although graphical representations of homology relationships are not new, their application to genetic distance computation with RNA-seq data is a contribution of the method described here. RNA-clique is designed to offer robustness in the presence of similar non-orthologous sequences. Unlike Cnidaria, RNA-clique explicitly identifies and compares orthologous transcripts using graph-based filtering. The graphs constructed by RNA-clique are also distinct from those of COG, which does not differentiate between orthologous and paralogous sequences. Identifying only orthologs allows RNA-clique to avoid overestimation of distances that could result from comparing paralogs or homeologs.

## Methods

The purpose of the algorithm developed is to compute values that quantify the similarity or distance among two or more individuals using sequences of RNA transcripts from those individuals captured with RNA-seq. The output of the algorithm is a matrix of values between 0 and 1 for each pair of individuals under consideration; we refer to these values as “genetic distances.” The genetic distance for a pair of individuals is interpreted as the degree of dissimilarity between the individuals’ genomes. The output distance matrix is then useful for downstream analyses such as genotyping and phylogenetics—the distances may be used to distinguish individuals by genotype or infer evolutionary relationships. Requirements of the method were that it be applicable to RNA-seq data from organisms with large and complex genomes and that pairwise comparisons for genetic distance calculations be between orthologs only, and not involve comparisons of paralogs or homeologs (which occur in allopolyploid species).

We first describe in general terms how RNA-clique uses RNA-seq data to compute pairwise genetic distances in “Distance computation algorithm” section. Descriptions of the data with which we tested our method and the tests performed are presented in the following “Data and Tests performed” sections, respectively.

### Distance computation algorithm

#### Assembling transcriptomes and selecting top genes

Each “sample” is an RNA-seq dataset from an individual, and different samples may be from the same individual (biological replicates) or different individuals. As in gene expression studies, it is important to include biological replicates for each individual. The dataset from each sample is first assembled into a “transcriptome,” which consists of many assembled transcripts or isotigs and is partitioned into “isotig sets” (i.e., genes). Each isotig in an isotig set is assumed to represent a splice variant or an allelic variant from the same gene, and every isotig in a transcriptome is assumed to have an associated “*k*-mer coverage”, which quantifies the amount of sequence from the input sequence reads that contributes to the assembled isotig. The *k*-mer coverage of a gene is defined as the maximum *k*-mer coverage among the isotigs of that gene, and, after assembly, the top *n* genes are identified based on *k*-mer coverage.

#### Computing distance for a pair of samples

Distance computation for a pair of samples is described below. The next subsection (“Computing distances for multiple samples” section) describes modifications to this basic approach for computing pairwise distances among more than two samples.

The top *n* genes (see “Assembling transcriptomes and selecting top genes” section) from both samples are used as the query and subject sequences in two BLASTn searches [5, 6]. In the first search, the top *n* genes from the first sample are BLASTed against the top *n* genes from the second sample, and in the second search, the top *n* genes from the second sample are BLASTed against the top *n* genes from the first sample. The result of either BLAST search is a table (dataframe) representing high-scoring segment pairs (HSPs). Partial example results for forward and reverse HSPs are shown in Tables 1 and 2. Note that although what we refer to as HSPs are commonly known as “hits,” in the terminology used by NCBI BLAST+, a hit may consist of one or more HSPs. Each HSP (i.e., each row in the table) specifies a query gene ID, query isotig ID, subject gene ID, subject isotig ID, bitscore, number of identical nucleotides, length, and gaps for the alignment. The bitscore measures the quality of an alignment in a way that does not depend on the size of the database (in this case, the subject transcriptome) and thus can be used to compare HSPs from different BLAST searches.

**Table 1:** Example partial results for “forward” matches

qgene	qiso	sgene	siso	bitscore	nident	length	gaps
0	0	6	0	20185	11073	11141	13
3	0	1	0	28334	15414	15449	1
3	0	18	0	28300	15400	15400	0
25	0	1996	5	804	437	438	0
25	0	1996	4	804	437	438	0
58254	0	48727	0	627	494	560	45

Data in the example tables are based on real data for tall fescue, but some rows have been modified for the sake of illustration

**Table 2:** Example partial results for “reverse” matches

qgene	qiso	sgene	siso	bitscore	nident	length	gaps
6	0	0	0	20185	11073	11141	13
1	0	3	0	28334	15414	15449	1
8	1	12	0	1851	1791	2178	29
8	1	12	1	1851	1791	2178	29
8	1	19	0	1850	1790	2170	29
48727	0	58254	0	616	492	560	45

For both tables of HSPs, we select the top *N* HSPs for each query gene ID, where *N* is a positive integer and a configurable parameter of the algorithm. For this paper, we always use $N = 1$, though future work may explore other settings for this parameter. Results of selecting the top HSP of each query gene ID in the example are shown in Tables 3 and 4.

**Table 3:** Forward matches after selecting top *N* HSPs for each query gene

qgene	qiso	sgene	siso	bitscore	nident	length	gaps
0	0	6	0	20185	11073	11141	13
3	0	1	0	28334	15414	15449	1
25	0	1996	5	804	437	438	0
25	0	1996	4	804	437	438	0
58254	0	48727	0	627	494	560	45

**Table 4:** Reverse matches after selecting top *N* HSPs for each query gene

qgene	qiso	sgene	siso	bitscore	nident	length	gaps
6	0	0	0	20185	11073	11141	13
1	0	3	0	28334	15414	15449	1
8	1	12	0	1851	1791	2178	29
8	1	12	1	1851	1791	2178	29
48727	0	58254	0	616	492	560	45

Note that each row in both tables contains one gene ID from the first sample and one gene ID from the second sample. We rename the columns in both tables to reflect this. In the table for the first search, the query gene ID and subject gene ID become the sample 1 gene ID and sample 2 gene ID, respectively. In the table for the second search, the query gene ID and subject gene ID become the sample 2 gene ID and sample 1 gene ID, respectively. The example tables become Tables 5 and 6 after renaming.

**Table 5:** Forward matches after renaming columns

s1gene	s1iso	s2gene	s2iso	bitscore	nident	length	gaps
0	0	6	0	20185	11073	11141	13
3	0	1	0	28334	15414	15449	1
25	0	1996	5	804	437	438	0
25	0	1996	4	804	437	438	0
58254	0	48727	0	627	494	560	45

**Table 6:** Reverse matches after renaming columns

s1gene	s1iso	s2gene	s2iso	bitscore	nident	length	gaps
0	0	6	0	20185	11073	11141	13
3	0	1	0	28334	15414	15449	1
12	0	8	1	1851	1791	2178	29
12	1	8	1	1851	1791	2178	29
58254	0	48727	0	616	492	560	45

Then, we filter both lists of HSPs to include only HSPs for which there is an HSP in both lists with the same sample 1 gene ID and sample 2 gene ID. The rows of the two tables are then merged into a single table. Note that the resulting table has at least two rows with the same sample 1 and sample 2 gene ID (Table 7).

**Table 7:** Rows where matches exist in both directions with the same s1gene and s2gene

s1gene	s1iso	s2gene	s2iso	bitscore	nident	length	gaps
0	0	6	0	20185	11073	11141	13
0	0	6	0	20185	11073	11141	13
3	0	1	0	28334	15414	15449	1
3	0	1	0	28334	15414	15449	1
5	1	627	0	7142	3899	3915	0
5	2	627	0	7142	3899	3915	0
5	3	485	0	7000	3899	3915	0
6	0	3	3	23525	12813	12850	0
6	1	3	3	23525	12813	12850	0
58254	0	48727	0	616	492	560	45
58254	0	48727	0	627	494	560	45

Some additional rows are shown to illustrate later steps

We then select the row with highest bitscore for each pair of sample 1 and sample 2 IDs present in the concatenated table. The result is a table that maps each pair of sample 1 and sample 2 IDs to a single best bitscore for that pair of genes (Table 8). Note that we may keep multiple rows in the case of ties, but in such cases there will still be a unique best bitscore for each gene pair.

**Table 8:** Table 7 with only the rows with top bitscore per gene pair selected

s1gene	s1iso	s2gene	s2iso	bitscore	nident	length	gaps
0	0	6	0	20185	11073	11141	13
0	0	6	0	20185	11073	11141	13
3	0	1	0	28334	15414	15449	1
3	0	1	0	28334	15414	15449	1
5	1	627	0	7142	3899	3915	0
5	2	627	0	7142	3899	3915	0
5	3	485	0	7000	3899	3915	0
6	0	3	3	23525	12813	12850	0
6	1	3	3	23525	12813	12850	0
58254	0	48727	0	627	494	560	45

Finally, we select the row with highest bitscore for each sample 1 gene (Table 9). In the resulting dataframe, we interpret each row as the most likely ortholog in sample 2 of the gene in sample 1. Again, we may keep multiple rows in the case of ties. We refer to the resulting table as the gene matches table for the two samples.

**Table 9:** The gene match table for two samples, which is Table 8 with only the rows with top bitscore per sample 1 gene selected

s1gene	s1iso	s2gene	s2iso	bitscore	nident	length	gaps
0	0	6	0	20185	11073	11141	13
0	0	6	0	20185	11073	11141	13
3	0	1	0	28334	15414	15449	1
3	0	1	0	28334	15414	15449	1
5	1	627	0	7142	3899	3915	0
5	2	627	0	7142	3899	3915	0
6	0	3	3	23525	12813	12850	0
6	1	3	3	23525	12813	12850	0
58254	0	48727	0	627	494	560	45

The similarity between the two samples is then the sum of the number of identical nucleotides for all rows in the table divided by the sum of the difference between the alignment lengths and gaps for all rows in the table. Equivalently, in symbols, let $\iota _i$, $\lambda _i$, and $\gamma _i$ represent the number of identical nucleotides, alignment length, and total gap length, respectively, for the i^th^ row in the table. Then, the similarity *S* between the two samples is

$$\begin{aligned} S = \frac{\sum _{i=1}^{k} \iota _i}{\sum _{i=1}^{k} \lambda _i - \gamma _i} \end{aligned}$$

The distance (or dissimilarity) *D* between the two samples is then defined as $D = 1 - S$. Since $\iota _i$, $\lambda _i$, and $\gamma _i$ are counts of base pairs, the resulting similarity is a dimensionless ratio of base pairs.

#### Computing distances for multiple samples

Of course, one straightforward way to find pairwise distances for more than two samples would be to apply the above procedure for finding the distance between two samples for each possible pair of samples. Although such an approach would be simple, we anticipate that this approach would give “unfair” comparisons because the homologous genes used for the comparison differ among pairs of samples. To address potential fairness problems, we employ a graph-based algorithm to find a subset of orthologous genes shared by all samples.

**Fig. 2:**
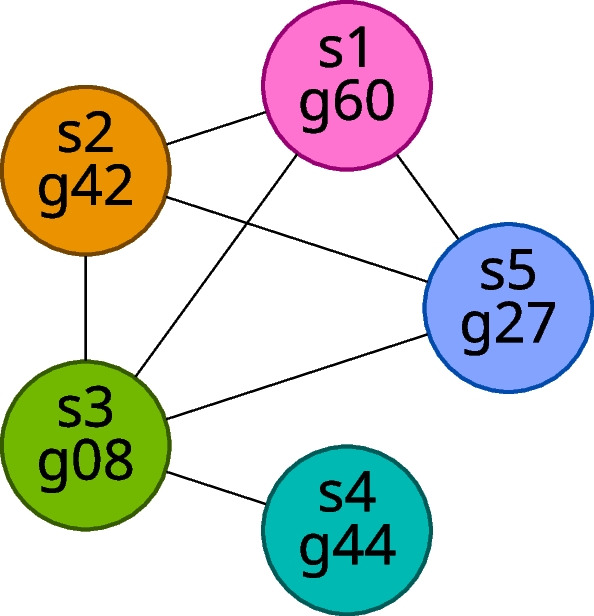
Example component of a gene matches graph. Vertex labels show sample ID and gene ID, and vertex colors indicate sample ID

We construct a graph, that is, a collection of vertices connected by edges, in which each vertex represents a gene in a particular sample; we can uniquely identify any vertex by its sample ID and gene ID. We draw an edge between two vertices if and only if the gene pair represented by the two vertices appears in the gene match table for the samples represented by the vertices. Intuitively, we can interpret an edge as indicating that the genes represented by its incident vertices are likely orthologs. We will refer to the resulting graph as the gene matches graph for the set of samples being considered. Figure 2 shows an example of a single connected component (a maximal set of vertices in which each pair of vertices is connected via a path of edges) from a gene matches graph.

**Fig. 3:**
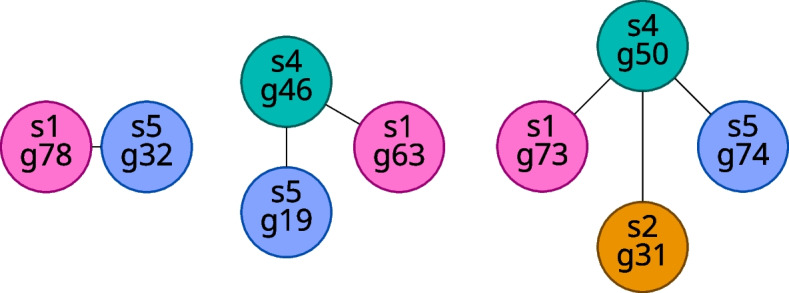
Examples of small components in the case of five samples

**Fig. 4:**
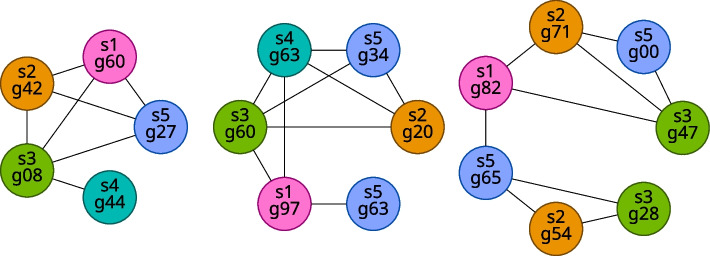
Examples of large components in the case of five samples

We can classify the components of the gene matches graph according to number of vertices. We define a **small component** as one with fewer vertices than there are samples, and, likewise, we define a **large component** as one with at least as many vertices as there are samples. Examples of small and large components for the case in which we have five samples are shown in Figs. 3 and 4, respectively.

**Fig. 5:**
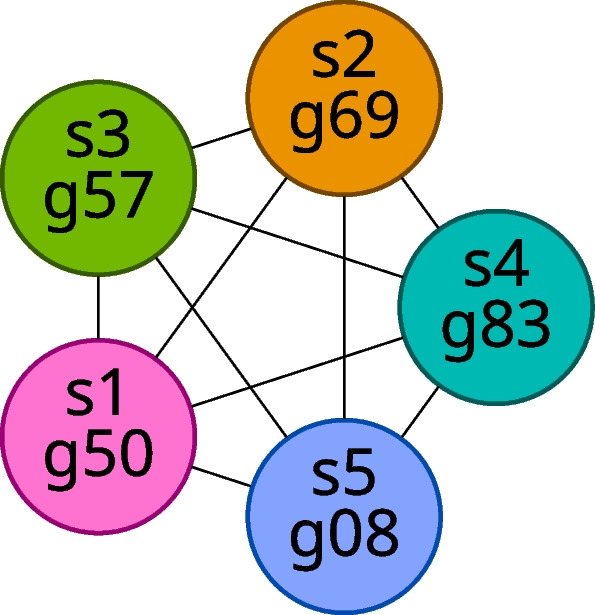
An example ideal component for five samples

Additionally, we classify some components as **ideal components**. We define an ideal component as a component that is a complete subgraph (that is, a clique, a subgraph with an edge between every pair of vertices) with exactly one gene from each sample. Note that this definition implies that an ideal component must also be a large component because an ideal component has exactly as many vertices as there are samples. An example ideal component (for the case of five samples) is shown in Fig. 5.

Since no two genes from the same sample may be connected by an edge, any complete component with exactly as many vertices as there are samples must have exactly one gene from each sample. Hence, we can equivalently consider an ideal component to be any component that is a complete subgraph and has as many vertices as there are samples.

The intent is that the vertices of an ideal component should represent genes for which exactly one ortholog is identified in every sample. Thus, in computing distances for multiple samples, we use only those rows of gene match tables whose sample 1 and sample 2 genes appear in some ideal component of the gene matches graph. The result of filtering the example data from Table 9 in this way is shown in Table 10.

**Table 10:** Table 9 restricted to gene pairs appearing in ideal components

s1gene	s1iso	s2gene	s2iso	bitscore	nident	length	gaps
0	0	6	0	20185	11073	11141	13
0	0	6	0	20185	11073	11141	13
3	0	1	0	28334	15414	15449	1
3	0	1	0	28334	15414	15449	1
5	1	627	0	7142	3899	3915	0
5	2	627	0	7142	3899	3915	0
6	0	3	3	23525	12813	12850	0
6	1	3	3	23525	12813	12850	0
58254	0	48727	0	627	494	560	45

### Data

Four sets of data were used for testing—one set of simulated transcriptomes and three sets of real data from past RNA-seq studies. Two of the datasets are from studies of the grass tall fescue (*Lolium arundinaceum*), and one is from a study of bluehead wrasse (*Thalassoma bifasciatum*), a teleost fish [7].

#### Tall fescue transcriptomes

Tall fescue, like many grasses (e.g., bread wheat) is “polyploid” due to an ancestry of hybridization between related species with intervening doubling of chromosome numbers. Having three diploid ancestors, tall fescue is hexaploid with a genome size estimated at $6x = 2C = 14.4 \text { Gb}$, over twice as large as the human genome [8]. The grass has a total of 42 chromosomes consisting of three homeologous sets, each with seven pairs of homologous chromosomes. For this reason, many genes—perhaps most—are represented by two or three homeologous sets, each having one or two (or at the population level, potentially more than two) homologous alleles [9]. Such polyploids are very common in certain plant families, and also in parthenogenic (or otherwise unisexual) animals and represent a special challenge to distinguish homologous versus homeologous gene relationships from mRNA or even genomic DNA sequence data. The tall fescue plant sources of the RNA-seq samples all derive from a single cultivar (‘Kentucky 31’), which in turn derives from a single ecotype—that is, all samples are descended from plants collected at the same location [2, 10]. The species is an obligate outcrosser, so each original plant represents a unique genotype. In the prior studies, the plants were divided and propagated as multiple clones, and the 16-sample dataset derives from multiple clones of each of four genotypes (plants). In some cases, clones were treated to eliminate the symbiotic fungus (endophyte) *Epichloë coenophiala*, and endophyte status ($+$ or −) is tracked in our analysis.

The RNA-seq reads were publicly available on NCBI’s Sequence Read Archive (SRA) and were assembled using the rnaSPAdes mode of version 3.15.5 of the SPAdes assembler [11]. We expected distances between samples from the same set of clones to be much smaller (ideally, zero) than distances between samples in different sets. The information for the samples used is summarized in Table 11.

**Table 11:** Metadata for 16 tall fescue samples used in testing

SRA accession	Genotype	Endophyte	Sequence reads	Genes	Transcripts
SRR2321388	CTE46	Infected	29193663	176482	205560
SRR2321387	CTE46	Infected	33344784	226633	262579
SRR2321386	CTE46	Infected	27762703	201459	236731
SRR2321385	CTE46	Minus	33335095	182658	219257
SRR2321384	CTE46	Minus	34098202	182206	218546
SRR2321383	CTE46	Minus	32274845	200285	242146
SRR8003761	CTE27	Infected	27287770	185253	218041
SRR8003753	CTE27	Infected	22208431	171965	198543
SRR8003754	CTE27	Infected	30235045	211796	248992
SRR8003762	CTE27	Minus	27057013	184385	217743
SRR8003755	CTE27	Minus	24162931	185532	216966
SRR8003756	CTE27	Minus	33508401	205484	247884
SRR7990321	FATG4	Infected	27592079	156343	182922
SRR7990322	FATG4	Infected	23795326	143898	168813
SRR8003736	NTE	Infected	20259358	144306	160606
SRR8003737	NTE	Infected	21715734	139838	156312

**Fig. 6:**
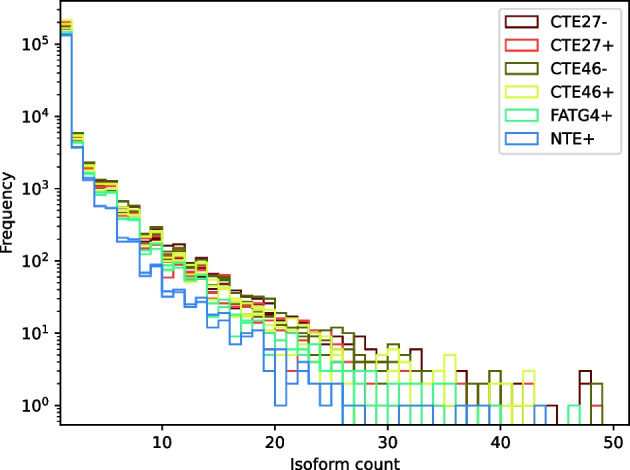
A histogram showing the frequency of isoform counts for genes in the 16 tall fescue samples. Note that the y-axis uses a logarithmic scale

rnaSPAdes may identify some transcripts as isoforms (or “isotigs”) of the same gene. Table 11 shows that the number of transcripts was much larger than the number of genes for each sample, but analyzing the frequency with which genes had one or more transcripts revealed that overwhelmingly most genes had very few isoforms (see Fig. 6).

RNA-seq data of four other samples available on the SRA (Table 12) were also used only in a test of the effect of the parameter *n* on the number of large components and ideal components in the gene matches graph (“Parameter tests” section). These reads were likewise assembled into transcriptomes with rnaSPAdes 3.15.5.

**Table 12:** Metadata for four tall fescue samples used for parameter *n* test only

SRA accession	Sequence reads	Genes	Transcripts
SRR6847395	64912332	219498	272178
SRR6847398	57343101	200289	247507
SRR6847396	79731246	306329	384056
SRR6847401	66194575	207645	260143

#### Bluehead wrasse transcriptomes

RNA-seq data for the bluehead wrasse originated from a study of gene expression in two tissue types involved in functional sex change [7]. In bluehead wrasse, individuals can undergo sex change in response to social cues. Specifically, loss of the terminal phase (TP) male from a bluehead social group can cause females and smaller initial phase males to become TP males. The original study of Liu et al. utilized the sequences of RNA extracted from the gonads and brain (midbrain/forebrain) of 12 individuals captured from patch reefs near Key Largo, Florida. The latter tissue type was used because of its role in social decision making.

Like the tall fescue RNA-seq reads, the bluehead wrasse reads were available from the SRA. Each tissue sample from each individual has been assigned an accession in the NCBI BioSample database and a sample ID incorporating the a numeric identifier for the individual and a letter, “G” or “F”, denoting tissue type “gonad” or “midbrain/forebrain”, respectively (Table 13). Each sample was associated with two SRA experiments, and, in turn, each experiment was associated with a single SRA run [7]. Each SRA run was associated with paired-end RNA-seq reads. Using the rnaSPAdes mode of SPAdes 3.15.5, we assembled all RNA-seq reads associated with each sample into a single transcriptome for that sample. Reads from different SRA experiments were provided as separate libraries to SPAdes. One SRA experiment, SRX1176335, belonging to BioSample SAMN04009766, was associated with some additional reads that were treated as unpaired reads from the same library as the others belonging to the experiment.

**Table 13:** Metadata for 24 bluehead wrasse samples used for distance tests only

Sample ID	BioSample accession	Tissue type	Individual ID
TBK12_1_F	SAMN04009769	Forebrain/midbrain	1
TBK12_1_G	SAMN04009770	Gonad	1
TBK12_6_F	SAMN04009771	Forebrain/midbrain	6
TBK12_6_G	SAMN04009772	Gonad	6
TBK12_8_F	SAMN04009773	Forebrain/midbrain	8
TBK12_8_G	SAMN04009774	Gonad	8
TBK12_15_F	SAMN04009781	Forebrain/midbrain	15
TBK12_15_G	SAMN04009782	Gonad	15
TBK12_18_F	SAMN04009783	Forebrain/midbrain	18
TBK12_18_G	SAMN04009784	Gonad	18
TBK12_50_F	SAMN04009785	Forebrain/midbrain	50
TBK12_50_G	SAMN04009786	Gonad	50
TBK12_52_F	SAMN04009763	Forebrain/midbrain	52
TBK12_52_G	SAMN04009764	Gonad	52
TBK12_114_F	SAMN04009765	Forebrain/midbrain	114
TBK12_114_G	SAMN04009766	Gonad	114
TBK12_117_F	SAMN04009775	Forebrain/midbrain	117
TBK12_117_G	SAMN04009776	Gonad	117
TBK12_118_F	SAMN04009767	Forebrain/midbrain	118
TBK12_118_G	SAMN04009768	Gonad	118
TBK12_120_F	SAMN04009777	Forebrain/midbrain	120
TBK12_120_G	SAMN04009778	Gonad	120
TBK12_121_G	SAMN04009780	Gonad	121
TBK12_121_F	SAMN04009779	Forebrain/midbrain	121

#### Simulated transcriptomes

**Fig. 7:**
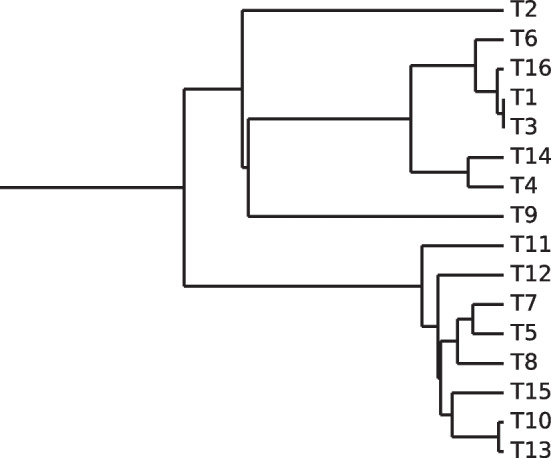
A tree showing the “ground-truth” phylogeny for the 16 simulated transcriptomes

We used the birth-death model implemented in the DendroPy Python library to generate a random phylogenetic tree with 16 extant taxa [12]. For the birth-death model, we used a birth rate of 1 and a death rate of 0.5; the simulation was allowed to continue until there were exactly 16 extant taxa. The taxa were labeled using the default scheme in DendroPy—i.e., a taxon’s label is simply “T” followed by the index of the taxon. The tree resulting from this simulation is shown in Fig. 7.

Using the same library, we generated random root state sequences for 50000 simulated transcripts. Transcript lengths were drawn randomly from the frequency distribution of transcript lengths for the 16 tall fescue transcriptomes—that is, the probability of choosing a transcript length was proportional to the number of transcripts with that length among the 16 tall fescue transcriptomes. For each position in a transcript, the base at that position was selected uniformly at random from the set of four DNA bases. (This is the default behavior in DendroPy’s nucleotide character evolution model.) The count of transcripts, 50000, was selected based on the results of the tests determining the effects of the parameter *n* on the number of ideal components, described in “Parameter tests” section.

We used the HKY85 model with an evolution rate of 0.01 to simulate evolution of these base transcripts over the previously generated phylogenetic tree. The value 0.01 was selected after it was determined that the initially selected value 0.1 was too high for BLAST to be able to identify orthologs. We obtained 50000 sets of orthologous transcripts, each containing one transcript per extant taxon.

### Tests performed

For all tests described in the following sections, the parameter *N* (the number of top HSPs to select for each query gene ID after the initial BLASTn searches in both directions) and the BLASTn *e*-value cutoff were fixed. The settings for these parameters were selected at the outset of testing. *N* was set to 1 to avoid matching non-orthologous genes, and the *e*-value cutoff was fixed at $10^{-99}$ to ensure only homologous sequences were reported by BLASTn.

#### Parameter tests

A number of tests were performed to determine the effects of certain parameters on the gene matches graph. Specifically, we tested the effects of the parameter *n* (the number of genes selected) and the number of samples *s* on the number of large components and the number of ideal components. To accomplish this, we ran RNA-clique for various values of these parameters. For *n*, this was accomplished by directly setting this value of this parameter at the beginning of each run of RNA-clique. For *s*, we ran RNA-clique with various sized subsets of samples. In all tests, after each run of RNA-clique, the number of ideal components and large components in the gene matches graph was recorded.

For both the four-sample set and the 16-sample set, we tested the effect of varying parameter *n*, whereby we select the top *n* genes based on *k*-mer coverage (“Assembling transcriptomes and selecting top genes” section). We reasoned that genes with lower *k*-mer coverage are less likely to form ideal components, so that the number of ideal components should plateau at higher values of *n*. Greatly exceeding the number of genes required to reach that plateau would contribute to computation time with little or no gain of usable data for the subsequent distance comparisons. For the set of four tall fescue samples (Table 12), we ran RNA-clique with settings of the parameter *n* varying from 1000 to 306329 (the maximum number of genes among the four samples) in steps of 1000. For the set of 16 tall fescue samples (denoted ${\mathcal {F}}_{16}$ in this section; Table 11), we ran RNA-clique with a different sequence of parameter settings for *n*; this sequence of settings are the *x*-axis coordinates of the points in Fig. 9. We used this sequence for the set of 16 tall fescue samples because the sequence increases exponentially, has easily readable values, and has many fewer elements than the sequence used for the set of four tall fescue samples. The second of these properties was important to capture the relationship between *n* and the number of components of each type for small values of *n*, and the last property was important for saving time since running RNA-clique requires more time for larger sets of samples. For both sets of samples, and for each setting of *n*, the number of ideal components and large components in the gene matches graph resulting from running RNA-clique with that setting was recorded, and these pairs of values were plotted to illustrate the relationships between the variables.

For the set of 16 tall fescue samples, we also tested the effect of the number of samples (i.e., the parameter *s*) on the counts of each type of component in the resulting gene matches graph by running RNA-clique with subsets of various size. Of course, for $0< s < 16$, we have more than one subset ${\mathcal {S}} \subset {\mathcal {F}}_{16}$ such that $|{\mathcal {S}}| = s$ (that is, the number of elements in *S* is *s*), and, moreover, for $0< s < 15$, there exist ${\mathcal {S}} \subset {\mathcal {F}}_{16}$ and ${\mathcal {T}} \subset {\mathcal {F}}_{16}$ such that $|{\mathcal {S}}| = |{\mathcal {T}}| - 1 = s$ and ${\mathcal {S}} \not \subset {\mathcal {T}}$. Hence, testing the effect of *s* on the component counts by independently selecting a random subset of size *s* from ${\mathcal {F}}_{16}$ for each value of *s* tested could be a flawed approach.

Instead of independently selecting random subsets of size *s* for each value of *s*, we first selected a permutation of the elements of ${\mathcal {F}}_{16}$. We then used size *s* prefixes of the permutation—that is, the first *s* elements of the permutation—as our subsets of size *s*. Using such prefixes ensured that each subset tested was a superset of the last—that is, the subset used for $s + 1$ was always a superset of the subset used for *s*. We used this prefix approach for our first set of sample count tests. Specifically, we applied the prefix approach for a permutation in which samples were sorted by genotype and a permutation in which samples were interleaved by genotype. For each of these tests, we used $n = 50000$; the selection of this value for *n* was informed by the results of our tests with ${\mathcal {F}}_{16}$ observing the effect of *n* on component counts. For each prefix of both permutations, we ran RNA-clique, and, again, the number of large and ideal component counts were recorded. The purpose of the genotype-interleaved and genotype-ordered tests was to allow us to see whether the ideal component count drops more dramatically when a sample with a new genotype is added.

Prefix tests cannot address the problem that there are many possible subsets of *s* from ${\mathcal {F}}_{16}$, and, hence, they cannot fully capture the relationship between number of samples and component counts. To address this shortcoming, subsets of ${\mathcal {F}}_{16}$ were sampled using a “fair” strategy that tries subsets selected uniformly at random from subsets of a specific size and tries to spend the same amount of time on each size (i.e., each value of *s*). Since computing the gene matches graph generally takes more time for larger values of *s*, the fair strategy can initially try more subsets for smaller values of *s*. Since the number of combinations ${16 \atopwithdelims ()s}$ is increasing up to $s = 8$, this trend would not continue indefinitely; we would eventually exhaust all subsets for smaller values of *s*. For each subset ${\mathcal {S}}$ tried, we also varied values of *n*, but only the data for the case where $n = 50000$ are reported and discussed here. For each subset of size *s* and each value of *n*, we ran RNA-clique and recorded the number of large and ideal components. For each subset of size *s*, we plotted the number of large components and ideal components to observe the relationship between *s* and the number of each kind of component. Using this fair sample count approach, we tested a total of 606 subsets of varying sizes. 

#### Distance tests

For the set of 16 tall fescue samples, the set of 24 bluehead wrasse samples, and the set of 16 simulated transcriptomes, pairwise distance matrices were estimated. In all tests, we set the parameter $n = 50000$. We visualized the distance matrices as heatmaps and principal coordinates analysis (PCoA) plots, and phylogenetic analysis employed the neighbor-joining algorithm implemented in Biopython’s Phylo module [13].

*Distance tests with Cnidaria*

The distance tests for the set of 16 tall fescue samples and the set of 24 bluehead wrasse samples were repeated using the existing method Cnidaria instead of RNA-clique. Although Cnidaria can use either raw RNA-seq data or assembled transcriptomes, the distance tests were only performed using the assembled transcriptome mode. The distance test for the set of bluehead wrasse samples was also repeated using a hybrid approach in which the graph-based filtering of RNA-clique was first used to select those genes with orthologs in all samples, and the resulting orthologs were provided as input to Cnidaria.

#### Resource usage tests

We measured the time and memory usage of both RNA-clique and Cnidaria for varying values of *n*, *s*, and *j*, the number of parallel jobs, using the set of 16 tall fescue samples. Because Cnidaria may be executed on either raw RNA-seq reads or assembled transcriptomes, we tested both configurations. We also calculated the resource usage for assembling the 16 tall fescue sample transcriptomes; a fair comparison between Cnidaria in RNA-seq read mode with either method in transcriptome mode should account for time needed to assemble reads into transcriptomes. Since resource usage depends on the quantity of input data, the top *n* genes were selected at the beginning of both the RNA-clique and transcriptome-based Cnidaria tests. Although selection of the top *n* genes is not part of the original Cnidaria method, it was necessary to perform this step for Cnidaria to ensure a fair comparison. Since selection of the top *n* transcripts was necessary for both RNA-clique and one of the Cnidaria modes, we measured the selection step separately.

Time usage of a program was measured as the total wall-clock time elapsed during execution of the program. Memory usage was measured as the maximum sum resident set size (RSS) of the program’s process tree during execution. The RSS measures only virtual memory of the process that occupies space in RAM. The sum RSS for the process tree was polled every 0.1 s using the procpath utility.

Tests of resource usage for varying values of *n* used the full set of 16 tall fescue samples and set *n* to the same set of values used for the parameter *n* tests of the 16 tall fescue samples described in “Parameter tests” section. Since the top *n* genes cannot be computed for the unassembled RNA-seq reads, we did not run Cnidaria in RNA-seq mode for the parameter *n* resource usage tests. Tests of resource usage for varying values of *s* set $n = 50000$ and used prefixes of size 4 to 16 of a random permutation of the set of 16 tall fescue samples—this strategy was borrowed from the prefix tests in the parameter tests described in “Parameter tests” section.

Both RNA-clique and Cnidaria can benefit from parallelism by performing computation in multiple threads or processes. RNA-clique can select top genes, build BLAST databases and execute BLASTn searches in parallel. Cnidaria can build its Jellyfish *k*-mer databases using multiple threads and can also split its data into multiple “pieces” which may be analyzed in parallel [14]. For the tests of resource usage as *n* and *s* varied, no parallelism was utilized. We separately tested the effect of the number of parallel jobs *j* (i.e., threads or processes) on resource usage for both methods. In these parallelism tests, the full set of 16 tall fescue samples was used with the fixed parameter setting $n = 50000$. The number of parallel jobs was varied from 1 to 16.

Resource usage tests for assembly were performed with SPAdes (version 3.15.5). SPAdes was allowed to allocate up to 120 GB of memory (though no assembly required that amount of memory). Although assembly can benefit from paralellism by running multiple assemblies in parallel or increasing the number of threads to use with SPAdes, neither option was utilized—only a single assembly was run at a time with one thread.

All tests assessing resource usage were performed on a computer with an AMD Ryzen 9 3950X CPU @ 2.2 GHz. The CPU had 16 physical cores, and frequency boosting up to 4.761 GHz was enabled. The computer had 117 GiB of RAM, and all data were read from and written to a PCIe 4.0 NVMe drive.

## Results

### Parameter tests

**Fig. 8:**
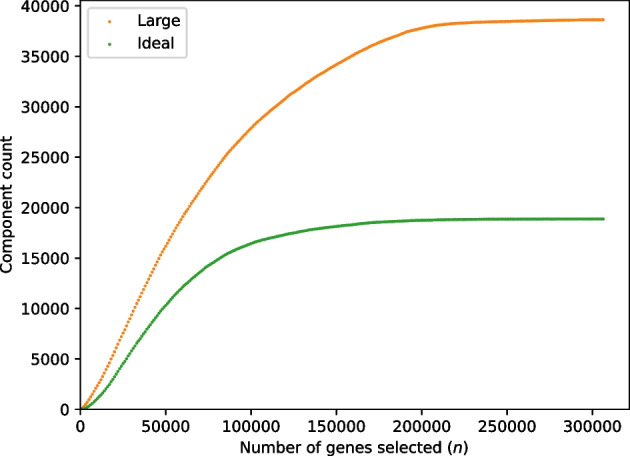
Large component and ideal component counts in the gene matches graph as the parameter *n* changes for the set of four tall fescue samples

**Fig. 9:**
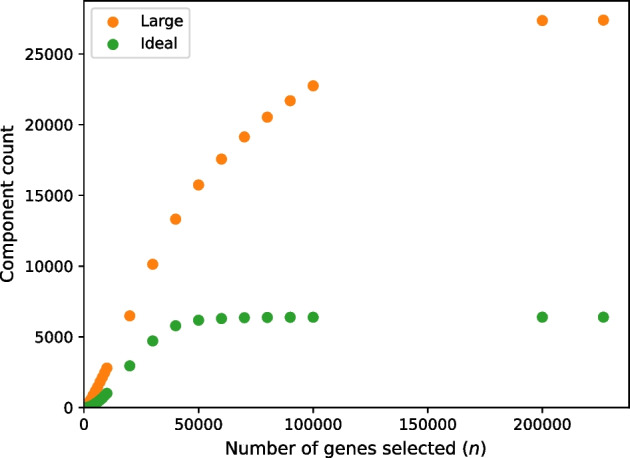
Large component and ideal component counts in the gene matches graph as the parameter *n* changes for the set of 16 tall fescue samples

Plots displaying gene matches graph component counts for varying values of *n* in the set of four tall fescue samples and the set of 16 tall fescue samples are shown in Figs. 8 and 9, respectively. Counts for both component types almost always increased with *n*. The *rate* of increase in ideal components increased for small values of *n* but decreased for large values of *n* until the counts of ideal components leveled off.

**Fig. 10:**
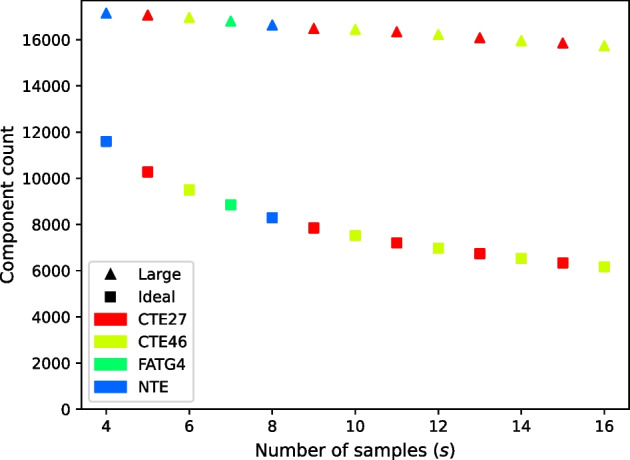
Large components and ideal components for prefixes of varying size *s* from a permutation of the 16 tall fescue samples in which samples are interleaved by genotype. Marker shapes denote the kind of component counted. Colors indicate the genotype of the last sample in the prefix

**Fig. 11:**
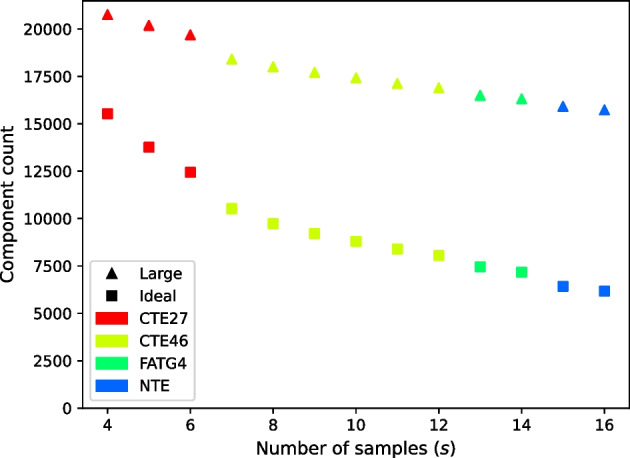
Large components and ideal components for prefixes of varying size *s* from a permutation of the 16 tall fescue samples in which samples are ordered by genotype. Marker shapes denote the kind of component counted. Colors indicate the genotype of the last sample in the prefix

For our genotype-ordered permutation, we found that adding a sample of a genotype not already present resulted in slightly greater decrease in ideal components than adding a sample with a genotype already present (Figs. 10 and 11).

**Fig. 12:**
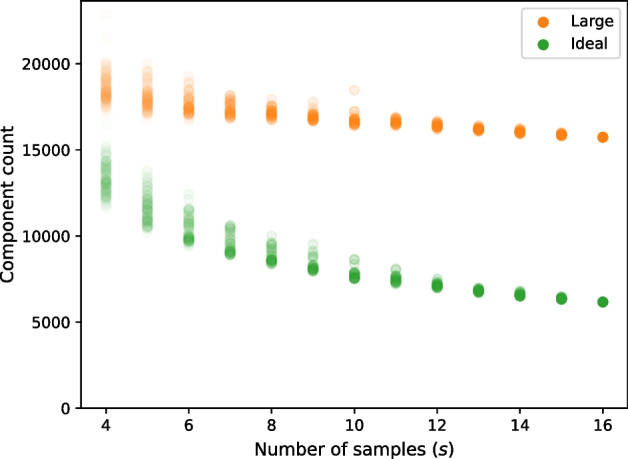
Large component and ideal component counts for randomly selected subsets of size *s*. The opacity of each point shown for *s* samples is inversely proportional to the number of subsets of size *s* tested

Figure 12 shows component counts for many randomly selected subsets of each size *s* from the set of 16 tall fescue samples. The variances in both component types decreased as *s* increased. (Note that there were fewer results for larger values of *s*, both because $$\left( {\begin{array}{c}16\\ s\end{array}}\right)$$, 16 choose *s,* is decreasing for $s > 8$ and because tests become more time consuming as *s* increases, requiring the “fair” strategy to attempt fewer tests for large *s*.)

### Distance tests

**Fig. 13:**
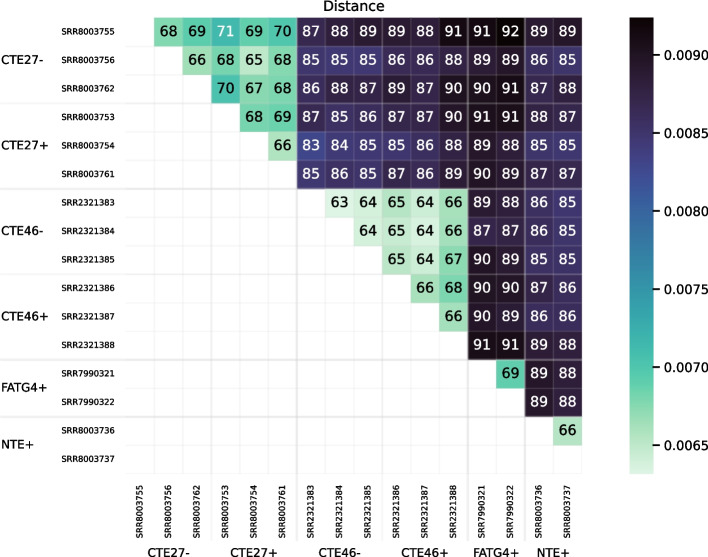
Heatmap showing distance between samples in the set of 16 tall fescue samples. A scale mapping colors to distance values is shown on the right, and each cell of the heatmap is annotated with its distance expressed in ten thousandths. Note that no diagonal is shown for this matrix

The heatmap in Fig. 13 visualizes the distance matrix obtained for the set of 16 fescue samples. The samples are ordered by genotype and endophyte status on both axes. Distances measured ranged from $0.0063$ to $0.0092$ between samples.

Figure 1 visualizes the distance matrix for the 16 tall fescue samples using PCoA, in which samples of the same genotype formed clusters. Generally, the distance between two samples of the same genotype was less than the distance between two samples of different genotypes. Although three samples each from two of the genotypes either possessed or lacked endophyte, little or no effect of endophyte was observed in the PCoA plot. (No additional separation was evident in a 3-dimensional PCoA, not shown.)

**Fig. 14:**
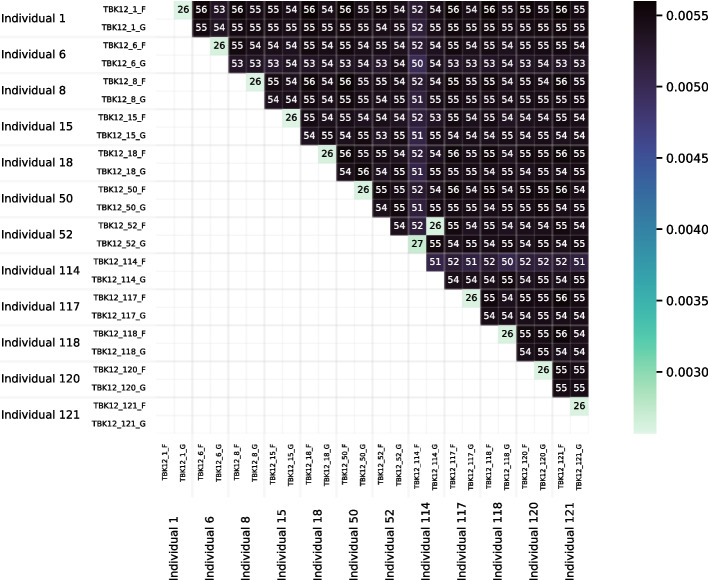
Heatmap showing distance between samples in the set of 24 bluehead wrasse samples. A scale mapping colors to distance values is shown on the right, and each cell of the heatmap is annotated with its distance expressed in ten thousandths. Note that no diagonal is shown for this matrix

Figure 14 is a heatmap visualizing the distance matrix for the set of 24 bluehead wrasse samples. The samples are ordered first by individual and then by genotype. Distances among the bluehead wrasse samples ranged from 0.0026 to 0.0056. For most samples, the closest sample was the other sample from the same individual. The exceptions were the individual 52 and individual 114 samples. The individual 52 forebrain was closest to the individual 114 gonad, and vice versa. Likewise, the individual 52 gonad was closest to the individual 114 forebrain, and vice versa. This stark result suggested that our method detected sample labeling errors.

**Fig. 15:**
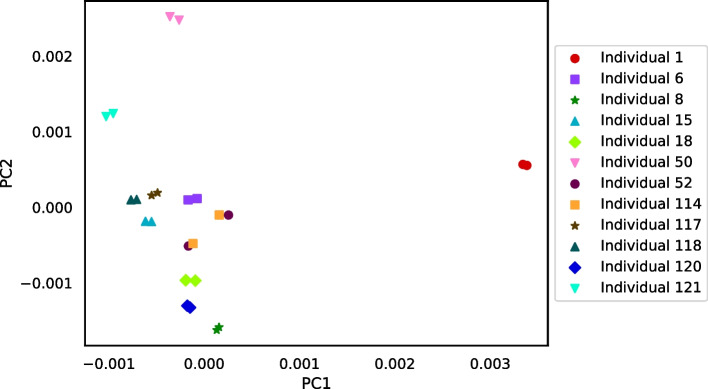
PCoA plot for the distance matrix of the 24 bluehead wrasse samples. Each point represents a sample, and color indicates the individual to which a sample was assigned in the SRA

The PCoA plot in Fig. 15 also visualizes the bluehead wrasse distance matrix. Although most samples were much closer to the other sample from the same individual than they were to any other sample, both individual 52 samples were closest to individual 114 samples, and both individual 114 samples were closest to individual 52 samples.

In the simulation study with 16 sets of sequences, the phylogenetic tree inferred from the calculated genetic distance matrix was topologically identical to the ground-truth tree in Fig. 7.

#### Distance tests with Cnidaria

**Fig. 16:**
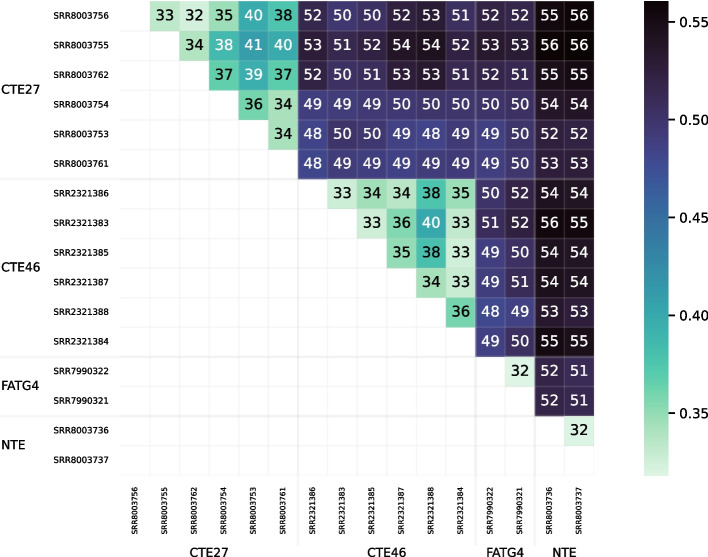
Heatmap showing distances computed by Cnidaria for the set of 16 tall fescue samples. A scale mapping colors to distance values is shown on the right, and each cell of the heatmap is annotated with its distance expressed in hundredths

Figure 16 visualizes the distance matrix computed with Cnidaria for the set of 16 tall fescue samples. Distances ranged from 0.32 to 0.56. Although the range differed from that for the distances computed using RNA-clique (Fig. 13), the two distance matrices showed a similar pattern. The distances between samples of the same genotype were lower than those between samples of different genotype in both matrices.

**Fig. 17:**
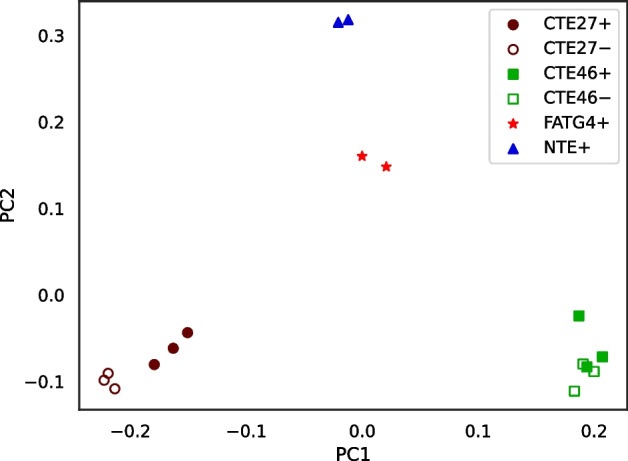
PCoA plot for the distance matrix computed with Cnidaria for the 16 tall fescue samples. Color and shape indicate genotype, and fill indicates endophyte status

Figure 17 is a PCoA plot created from the matrix in Fig. 16. As in the PCoA plot for the distance matrix computed using RNA-clique (Fig. 1), the samples clustered according to genotype, but the CTE27 and CTE46 clusters showed greater spread in the PCoA plot for the Cnidaria distance matrix.

**Fig. 18:**
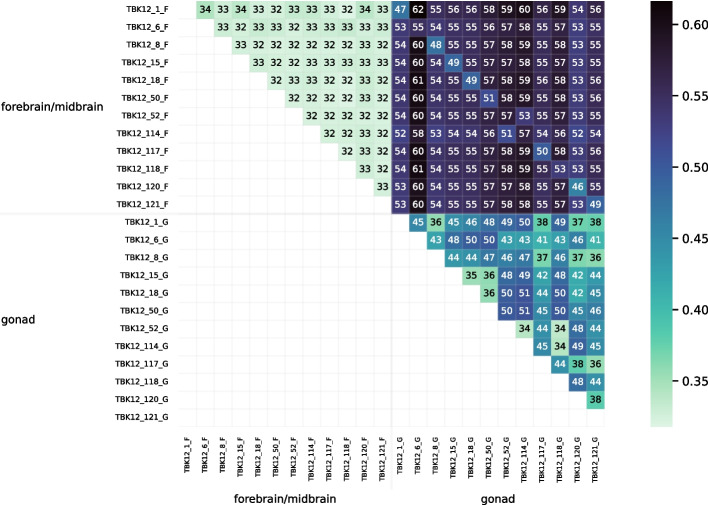
Heatmap showing distances computed with Cnidaria for the set of bluehead wrasse samples. A scale is shown to the right, and cells are annotated with distance values expressed in hundredths

The heatmap in Fig. 18 visualizes the distance matrix calculated by Cnidaria for the set of 24 bluehead wrasse samples. Unlike the samples in Fig. 14, those in Fig. 18 are ordered first by tissue and second by individual. Distances ranged from 0.32 to 0.62. Distances between samples of the same tissue type were generally estimated to be smaller than those between samples of different tiissue type. Although the lowest distances were not between samples from the same individual (as they were in Fig. 14), the values on the diagonal of the upper-right quadrant of the matrix (the submatrix consisting of distances between samples of different tissue type) showed that distances between samples from the same individual tended to be lower than distances between other pairs of samples from different tissue types.

**Fig. 19:**
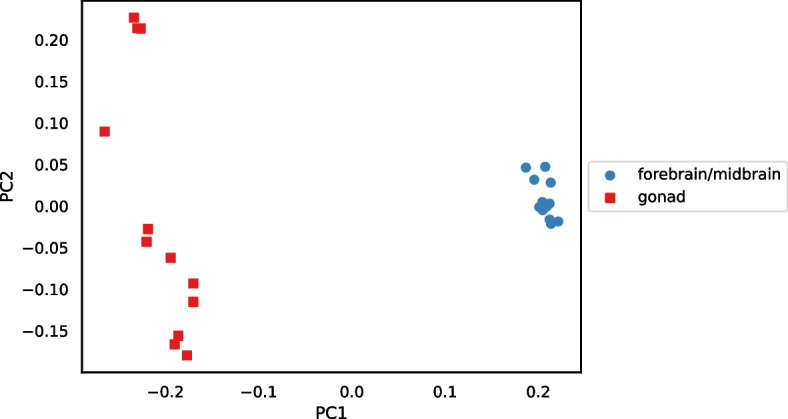
PCoA plot for the distance matrix computed with Cnidaria for the 24 bluehead wrasse samples. Color and shape denote tissue type

Figure 19 is a PCoA plot for the Cnidaria bluehead wrasse distance matrix. All forebrain/midbrain samples formed a cluster, but the gonad samples were apparently spread out into multiple small clusters along the second principal component axis. Nevertheless, the gonad samples were near each other on the first axis, and all gonad samples were distant from the forebrain/midbrain cluster.

**Fig. 20:**
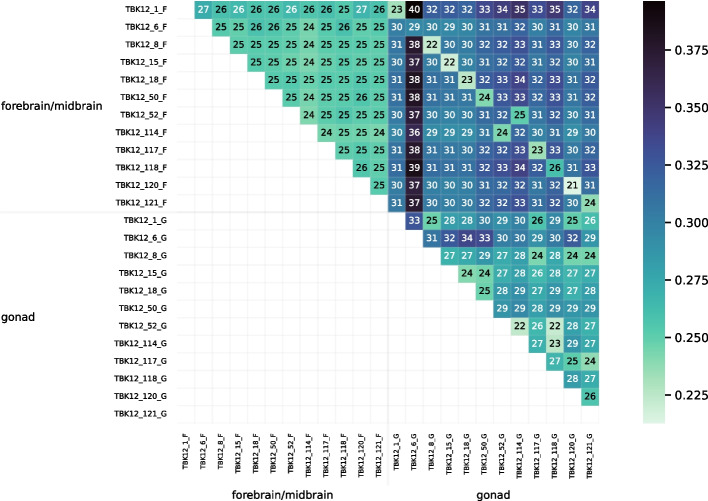
Heatmap showing distances computed with Cnidaria for the set of bluehead wrasse samples, after using RNA-clique to filter transcripts so that only genes in ideal components are included. A scale is shown to the right, and cells are annotated with distance values expressed in hundredths

**Fig. 21:**
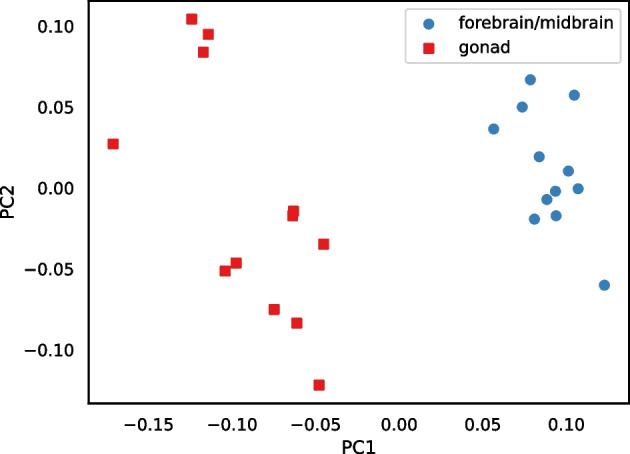
PCoA plot for the distance matrix computed with Cnidaria for the 24 bluehead wrasse samples, after using RNA-clique to filter transcripts so that only genes in ideal components are included. Color denotes tissue type

The heatmap in Fig. 20 visualizes the distance matrix obtained with the combined RNA-clique and Cnidaria approach (using RNA-clique to select genes with orthologs in all samples) for the set of 24 bluehead wrasse samples. As in Fig. 18, samples were sorted by tissue type and individual. Samples of the same tissue type were typically less distant than samples of different tissue types, but the difference between tissue types was less extreme than that observed in Fig. 18. Moreover, for any given sample, the best match was often the other sample from the same individual. Figure 21 is a PCoA plot for the distance matrix computed for the 24 bluehead wrasse samples using the hybrid approach. Although clusters were denser in Fig. 19 than in Fig. 21, there nevertheless remained a clear separation between forebrain/midbrain and gonad samples in the latter plot.

### Resource usage tests

**Fig. 22:**
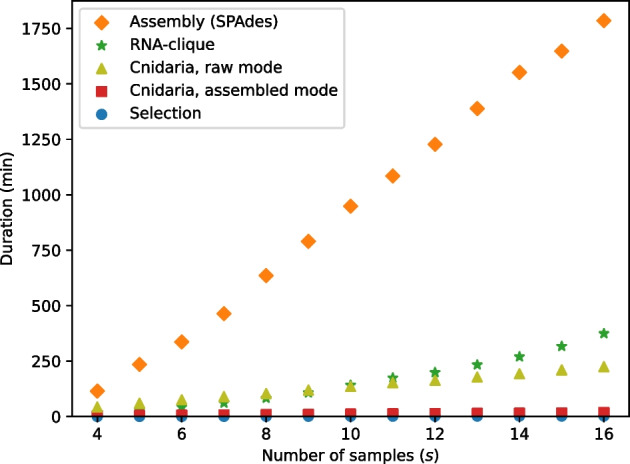
Execution times for running parts of various RNA-seq to distance matrix pipelines with varying numbers of samples and one parallel job. “Selection” is the script that selects the top $n = 50000$ genes from each of the transcriptomes, which was executed before RNA-clique or Cnidaria in its assembled mode

Tests of the effect of sample count (*s*; Fig. 22) showed that, when only one parallel job was used, transcriptome assembly with SPAdes was the most time-consuming process in any of the pipelines for obtaining genetic distance matrices from RNA-seq data. The “Selection” process represented the selection of top *n* genes by *k*-mer coverage (“Assembling transcriptomes and selecting top genes” section), which was used in both the RNA-clique and assembled-mode Cnidaria pipelines. Times shown for RNA-clique and assembled-mode Cnidaria did not include the selection time. RNA-clique was the second or third most time-consuming process, depending on *s*. RNA-clique’s running time was approximately quadratic in *s* for the values of *s* tested; all other programs were roughly linear in *s*. Applying quadratic least-squares regression to the running times for RNA-clique produced a model ($r^2 = 0.9984$) of RNA-clique’s running time in seconds as a function of *s*, $t_{\text {R}}(s) = 3263.683s^2 + 10541.403s + 8169.31$. Likewise, applying linear least-squares regression to the running times for Cnidaria in assembled mode produced a model ($r^2 = 0.9995$) of Cnidaria’s running time in seconds as a function of *s*, $t_{\text {C}}(s) = 414.866s + 672.287$.

**Fig. 23:**
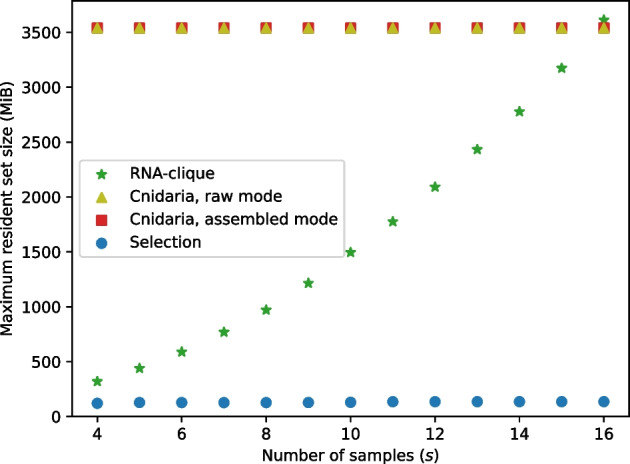
Maximum RSS for running parts of various pipelines with varying numbers of samples and one parallel job

Maximum RSS (memory usage) for varying values of *s* is shown in Fig. 23. Although maximum RSS values for SPAdes assembly were recorded, the values were not included in the plot because they were much higher (as large as 14.66 GiB) than those for the other programs. Both modes of Cnidaria had a maximum RSS of 3.46 GiB, independent of the value of *s*. The selection process maximum RSS increased in steps due to differences in transcriptome size among the samples but never exceeded 135.75 MiB. Although memory usage for RNA-clique was lower than that for Cnidaria for $s < 16$, the maximum RSS of RNA-clique scaled roughly quadratically with *s*. Applying quadratic least-squares regression to the maximum RSS of RNA-clique produced a model ($r^2 = 0.9999$) of RNA-clique’s memory usage in MiB as a function of *s*, $m_{\text {R}}(s) = 477.319s^2 + 1647.475s + 1480.589$.

**Fig. 24:**
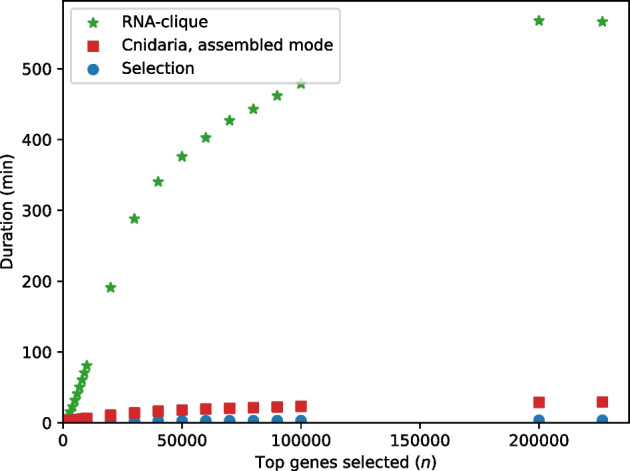
Execution times for running parts of the RNA-clique and assembled-mode Cnidaria pipelines with varying values for *n*, the number of top genes to select

Figure 24 shows the execution times of the selection process, RNA-clique, and Cnidaria for various settings of the parameter controlling the number of top genes to select by *k*-mer coverage, *n*. Selection required very little time—always less than 150 s. The rate of change in running times in Fig. 24 decreased with *n*, causing the running times to level off.

**Fig. 25:**
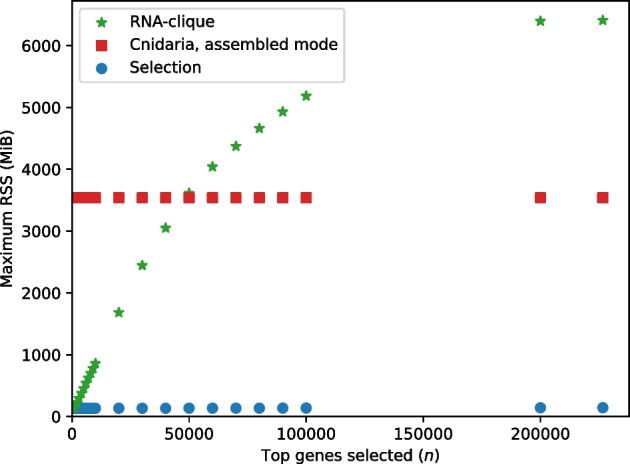
Maximum RSS for parts of the RNA-clique and assembled-mode Cnidaria pipelines with varying values for *n*, the number of top genes to select

Figure 25 shows the maximum RSS for the selection process, RNA-clique, and Cnidaria for varying values of *n*. As in the results measuring the effect of the number of samples *s* on maximum RSS, Cnidaria used no more than 3.46 GiB, regardless of parameter setting. The selection process maximum RSS increased slightly with *n*. The difference in memory usage for $n = 226633$ (the maximum setting of *n*) and for $n = 1000$ was only 9.5 MiB, a $7\%$ increase. The maximum RSS for RNA-clique likewise increases with *n* (and is generally much higher than the memory usage for selection), but the rate of change in maximum RSS for RNA-clique also decreases with *n*.

**Fig. 26:**
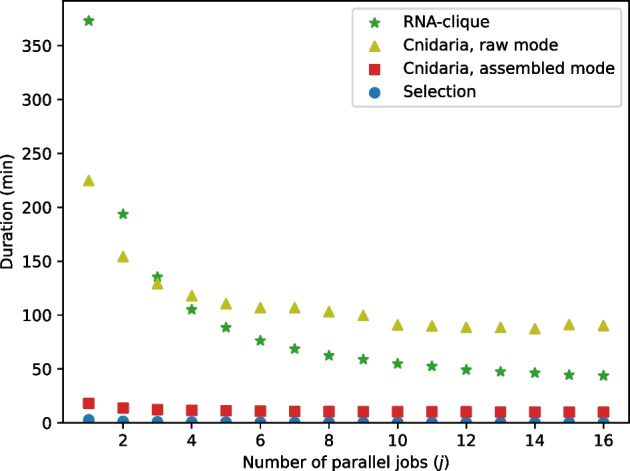
Execution times for parts of various RNA-seq to distance matrix pipelines with varying numbers of parallel jobs

Figure 26 shows results of the tests of the effect of parallelism (number of parallel jobs) on running times of the selection process, Cnidaria (both raw and assembled mode), and RNA-clique. All steps saw much improvement in running time with additional parallel jobs, especially RNA-clique, for which the duration decreased by 5.49 hours, $88.3\%$.

**Fig. 27:**
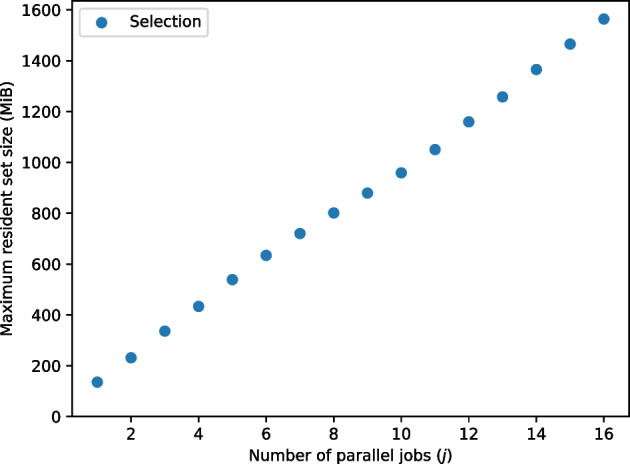
Maximum RSS for selection of top 50,000 genes with varying numbers of parallel jobs

For RNA-clique and Cnidaria, the maximum RSS increased very little (less than $0.3 \%$) as the number of parallel jobs increased. The memory needed by the selection process increased much more (around $1054 \%$) and increased roughly linearly with the number of parallel jobs. Only maximum RSS values for the selection process were included in Fig. 27.

## Discussion

Results of the distance tests on plant, animal, and simulated testbeds suggest that the method proposed, RNA-clique, gives sufficiently accurate pairwise distances to distinguish RNA-seq samples according to genotype or individual. Moreover, results of the parameter tests suggest that, for sufficiently similar individuals, enough genes were retained in ideal components on which to base the genetic comparisons. In the tall fescue 16-sample testbed, selecting the top $50000$ isotig sets by *k*-mer coverage gave more than $5000$ ideal components on which to base the distance calculations, and even with a very narrow range of inferred distances from approximately 0.9–$0.65\%$, samples from each genotype clearly clustered in a 2D PCoA plot. Likewise, for the bluehead wrasse 24-sample testbed, the samples clustered by individual. Comparisons with an alternative method, Cnidaria, favor RNA-clique. Although Cnidaria may be more scalable than RNA-clique, results from RNA-clique appear more reliable.

The PCoA plot for the 16 tall fescue samples (shown in Fig. 1) shows four distant and non-overlapping clusters of individuals—one for each genotype—and the heatmap confirms that the distances between individuals of the same genotype are always relatively low compared to distances between individuals of different genotypes (Figure S1). Nevertheless, RNA-clique detects noise in the form of small differences for each pair of individuals with the same genotype. Although plants with the same genotype should be clones, there are no two individuals for which the similarity is computed to be exactly 1. Of course, it is possible some detected differences between clones reflect actual mutations, but differences may also stem from various sources of error. One class of error that could affect the accuracy of the distances are sequencing errors. To understand the effect of a sequencing error on the calculated distance, suppose we have a pair of transcripts,$t_1$ and $t_2$, in one of the filtered gene matches tables, and, due to a sequencing error, $t_1$ has an erroneous base $b'$ where it should have *b* in the aligned region. Also, let *c* represent the corresponding base in $t_2$. (We assume there is no sequencing error at that position in $t_2$.) If $b = c$, then the erroneous base will appear as a spurious mismatch (a “false positive” difference). If instead $b \ne c$ and $b' = c$, the erroneous base will appear as a spurious identity (a “false negative” difference). Finally, if $b \ne c$ and $b' \ne c$, the erroneous base has no effect for that pair of transcripts—RNA-clique correctly counts it as a mismatch (a “true positive” difference).

Since the tall fescue samples are not haploid, homeologous transcripts may be a source of false differences. Specifically, if a genotype is heterozygous for some gene, but different alleles are captured in the transcriptomes of different clones, there is a risk that a transcript in one clone may erroneously be compared with a transcript that is not its true closest match in another clone. This kind of error would inflate the computed distances. Furthermore, even if all alleles are captured in the RNA-seq reads for all clones, there is a risk that the assembler may assemble reads belonging to different homeologs into a single isotig. If this happens inconsistently across different samples, the assembled transcripts for one clone may differ from those of another, and these differences could contribute to the computed distance between the clones. Such an assembly error could result in either overestimation or underestimation of distances.

The extent to which each of these factors contributes to the differences observed between samples of the same genotype may be explored in future research, and future refinements to RNA-clique may incorporate strategies for mitigating some factors. For example, sequencing and assembly errors may be detectable by consulting the original reads. Sequencing errors may appear as low-quality bases, and assembly errors could be detected by determining whether a detected difference between isotigs can be accounted for by an alternative assembly for one or both of the isotigs. In either case, differences identified as potentially spurious may be excluded from the distance calculation. Such refinements may be especially useful for very small or especially complex datasets. Although certain factors may lead to overestimation of distance in some circumstances, the results indicate that RNA-clique is effective at unambiguously grouping samples by genotype. The results of the tests with the set of 16 tall fescue samples also show that analyzing multiple samples per genotype is especially helpful for genotyping despite non-zero distances among clones since such distances are smaller than those between samples with different genotypes.

The results for the distance tests with the set of 24 bluehead wrasse samples show that RNA-clique can determine pairs of samples that belong to the same individual for at least 10 of the 12 individuals (20 of 24 samples). The method ostensibly gives some incorrect distances for individuals 52 and 114, but since RNA-clique identifies two pairs of closely related samples, both with one sample from each of the two individuals, we believe the error is likely caused by incorrect labeling of the samples. The labels for two samples of the same tissue type from individuals 52 and 114 may have been swapped in the SRA. That the swap is also evident in the results from Cnidaria (the upper-right quadrants of Figs. 18 and 20) suggest that the apparent mismatch is not a problem with RNA-clique. Furthermore, the results suggest that RNA-clique is a useful tool for verifying that RNA samples are correctly attributed to source individuals.

A comparison between the results obtained from RNA-clique and those obtained from Cnidaria shows that RNA-clique is as reliable or more reliable than Cnidaria, depending on the dataset. Results obtained by the two methods for the set of 16 tall fescue samples are very similar (though the scales of the distances are different). Nevertheless, the CTE27 and CTE46 clusters in the PCoA plot of the Cnidaria results (Fig. 17) are less dense than those in the corresponding plot of the RNA-clique results. Since we expect that samples of the same genotype should be identical, and, thus, should have no distance, this difference in the two plots may indicate that RNA-clique gives more accurate distances for these genotypes than does Cnidaria. In contrast, results obtained with the two methods for the set of 24 bluehead wrasse samples are markedly different. Almost all samples in the PCoA plot for RNA-clique (Fig. 15) form two-sample clusters according to individual as expected, but for Cnidaria, samples instead cluster according to tissue type (Fig. 19).

The Cnidaria method fails to identify the same genotypes in the bluehead wrasse dataset but succeeds with the tall fescue dataset. We considered as a possibility that the different tissues in the fish expressed sufficiently different sets of genes that most *k*-mers were specific to one or the other tissue. However, applying our ideal components strategy, which is meant to filter for true orthologs, does not qualitatively change the outcome. Another possibility is that, despite filtering for orthologs, the mRNA structures are sufficiently different due to, for example, alternative splicing [15, 16]. An alternatively spliced intron would lead to a number of unique *k*-mers comparable to the *k*-mer length, and those may dominate the distance calculation. In contrast, the distance used in RNA-clique is designed to avoid any effect of such differences in mRNA structure, and, perhaps for this reason, succeeds with the fish RNA-seq testbed.

Tests assessing the effect of parameter *n*, the number of top genes selected at the beginning of our method, on the number of ideal components in the gene matches graph reveal that there are diminishing returns for selecting more genes past a certain point (for the set of four tall fescue samples, we judge around $n = 20000$). For the set of 16 tall fescue samples, the difference between the count of ideal components at $n = 50000$ and at the maximum value for *n*, $n = 226633$, was only $216$; the increase in ideal components was only approximately $3.5 \%$. Therefore, for that study we judge $50000$ genes to be adequate for the analysis, and this represents much savings in time compared to exhaustive analysis.

Still, it is apparent that the extent to which we benefit (in terms of ideal component count) from selecting more genes depends on the number and kinds of samples we have, among other factors. The ideal component count increases little past $n = 50000$ for the set of 16 tall fescue samples, but there is still much that can be gained from selecting more than $50000$ genes in the set of four tall fescue samples. Future work may focus on modeling relationships between the ideal component count and the parameters *n* and *s*. Such a model might be useful for selecting appropriate values of *n* for new data if we can extrapolate predicted ideal component counts for large values of *n* from counts for smaller values of *n* for which the gene matches graph is faster to build.

Tests assessing the effect of the parameter *s* on the component counts show that although we obtain fewer ideal components on average as we increase *s* for a given value of *n*, we typically lose fewer components with each successive sample. Of course, some individuals in the set of 16 tall fescue samples are expected to be much more closely related than others, and the genotype-interleaved tests suggest that the similarity of a newly added sample to those previously considered can affect the decrease in ideal components. As we might expect, sufficiently dissimilar samples can cause the component count to drop to zero; we observed this with simulated data when we used a mutation rate of $0.1$ (data not shown) instead of the rate of $0.01$ we used for the tests described here. For very distantly related pairs of samples, there may be no BLAST hits at all; if such a pair is present among the set of samples, the gene matches graph will have no ideal components. For other sets of samples, there may be BLAST hits for every pair, but there may still be insufficient hits to form an ideal component. The effect of the samples’ similarity on the number of ideal components we obtain is a possible topic of future research that could be explored with additional simulated data. Specifically, observing how the number of ideal components we obtain varies as we change mutation rate may provide some insight into the relationship between similarity and ideal component count.

Results from the resource usage tests show that Cnidaria scales better than RNA-clique in terms of memory and time requirements, but RNA-clique’s resource usage is nevertheless sufficiently small to make it a practical method for handling moderately large sets of samples. Extrapolation with the regression models of running time and memory usage for RNA-clique ($t_{\text {R}}$ and $m_{\text {R}}$, respectively; “Resource usage tests” section) predicts that the computer used for the resource usage tests should be able to run RNA-clique with sets containing as many as 94 samples ($m_{\text {R}}(94) \le 117 \times 2^{10} < m_{\text {R}}(95)$), which would take 9.21 days with a single parallel job, or 25.85 hours with 16 parallel jobs. Provided enough memory, RNA-clique should be able to handle in one week sets of up to 82 samples with one parallel job ($t_{\text {R}}(82) \le 60^2 \times 24 \times 7 < t_{\text {R}}(83)$) or up to 239 samples with 16 parallel jobs ($(1 - 0.883) \times t_{\text {R}}(239) \le 60^2 \times 24 \times 7 < (1 - 0.883) \times t_{\text {R}}(240)$). To run RNA-clique with 82 samples would require 87.88 GiB, and to run RNA-clique with 239 samples would require 741.862 GiB. In contrast, Cnidaria should be able to handle very large sets of samples. The model for Cnidaria’s time usage ($t_{\text {C}}$; “Resource usage tests” section) suggests that Cnidaria should be able to handle in one week sets of up to 8747 samples with one parallel job ($t_{\text {C}}(8747) \le 60^2 \times 24 \times 7 < t_{\text {C}}(8748)$) or up to 15884 samples with 16 parallel jobs ($(1 - 0.449) \times t_{\text {C}}(15884) \le 60^2 \times 24 \times 7 < (1 - 0.449) \times t_{\text {C}}(15885)$).

Since RNA-clique appears to give more accurate results than Cnidaria, we believe RNA-clique should be the preferred method despite the latter method’s superior scalability. Still, the sources of error in Cnidaria’s distance matrix for the bluehead wrasse data are not fully known. Future work could focus on identifying these sources of error with the goal of improving the method or determining on which datasets Cnidaria can be used reliably.

### Future work

In addition to the possible future directions mentioned above, we would also like to further test our method using more synthetic data designed to simulate a wider range of scenarios. Since many commonly studied organisms are diploid or polyploid, we are especially interested in simulating hybridization of closely related taxa to investigate the effect that the presence of homeologs has on the accuracy of the calculated distances and correct matching of orthologs.

Although we think using simulated data would allow us to study more precisely how the number of samples *s* and samples’ relatedness affect ideal component count, we also plan to test this approach on data for larger—and perhaps more diverse—sets of organisms. Such tests may better inform us of the practical limitations of the method proposed.

Finally, we would like to explore the mathematical properties of the distances we compute and possibly refine our method based on our findings. Although we often describe the quantities we compute for each pair of samples as “distances”, we have not proven that our distance, as a function of a pair of transcriptomes, satisfies all properties one expects to hold for a distance metric. In particular, we believe the distance we compute may not necessarily be symmetric; i.e., computing the distance between sample *A* and sample *B* may not give the same result as computing the distance between sample *B* and sample *A*. We also have not proved that the triangle inequality holds; we do not know that the sum of distances from *A* to *B* and *B* to *C* are never less than the distance from *A* to *C*. We have yet to observe a counterexample for either property, but we have so far only tested RNA-clique on “realistic” data that may not be likely to explore cases in which these properties would be violated.

## Conclusion

Despite the aggressive filtering applied throughout the proposed method and the inherent limitations of considering only transcribed sequences, we find the approach described in this paper satisfactorily measures differences among closely related individuals in tests with both real and simulated data. Although the amount of data remaining after filtering depends on the number of samples used and the relatedness of those samples, the filtering process retains enough data to get useful pairwise distances for the testbed examples, provided that we set the parameter *n* sufficiently high.

The method has been tested on a hexaploid grass, a vertebrate animal, and simulated data with satisfactory results that suggest RNA-clique may be equipped to handle other organisms of practical interest that possess similarly complex genomes, including humans and many other animals. The method is not without some limitations. Applying RNA-clique to simulated data generated using a high mutation rate (data not shown) revealed that samples may be too distantly related to compare with this method. Likewise, there may be some datasets where samples are too closely related to distinguish above the noise. Comparisons for time and memory usage for RNA-clique versus Cnidaria suggests that the latter may sometimes be preferable for very large sets of samples with the caveat that Cnidaria may not produce as accurate results depending on the nature of the sample sets. Therefore, if the data set is too large for RNA-clique, it may be a useful strategy to check results of Cnidaria against results of RNA-clique on a subset of samples.

Although further work is required to determine how distantly or closely related the samples may be in order for RNA-clique to be practical, we nevertheless think that the results of our tests indicate the method proposed here is useful for generating pairwise distance matrices based on multiple RNA-seq datasets for a wide range of organisms and experiments.

### Supplementary Information

Supplementary file 1

## Data Availability

The tall fescue RNA-seq data analyzed during the current study are available from the NCBI Sequence Read Archive at https://www.ncbi.nlm.nih.gov/sra using the accessions provided in Tables 11 and 12. The bluehead wrasse data are likewise available from the Sequence Read Archive and may be found using the BioSample accessions provided in Table 13 The simulated transcriptomes analyzed during the current study are available from the corresponding author on reasonable request.
