# Supplementary material for: RNA-clique: a method for computing genetic distances from RNA-seq data
Source: BMC Bioinformatics. 2024 Jun 4;25:205. doi: 10.1186/s12859-024-05811-9 (PMC11149392; doi:10.1186/s12859-024-05811-9)
Supplement: Supplementary file 1 [file 12859_2024_5811_MOESM1_ESM.pdf]

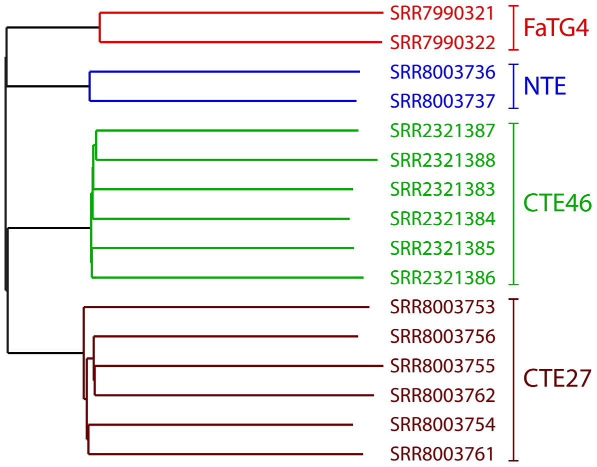

Fig. S1 Midpoint-rooted neighbor-joining tree constructed from the distance matrix for the set of 16 tall fescue samples. Label colors indicate genotype.

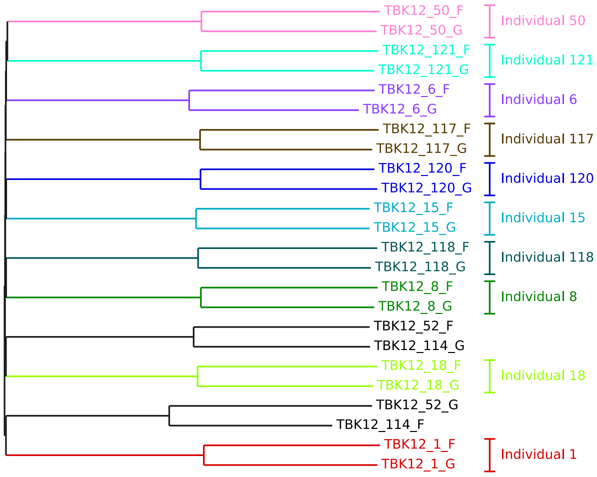

Fig. S2 Midpoint-rooted neighbor-joining tree constructed from the distance matrix for the set of 24 bluehead wrasse samples. Clade colors denote individual.

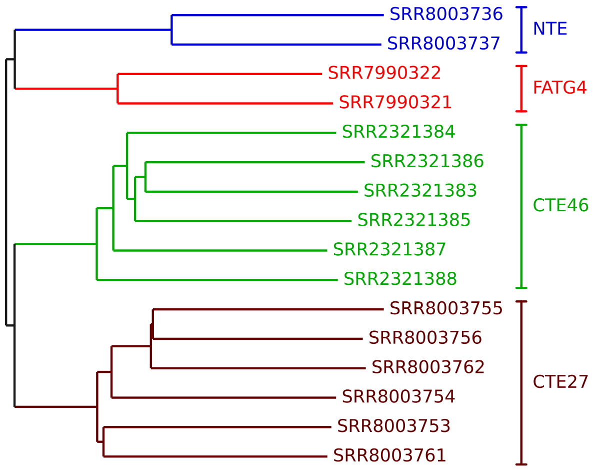

Fig. S3 Midpoint-rooted neighbor-joining tree constructed from the distance matrix computed with Cnidaria for the 16 tall fescue samples. Label colors denote genotype.

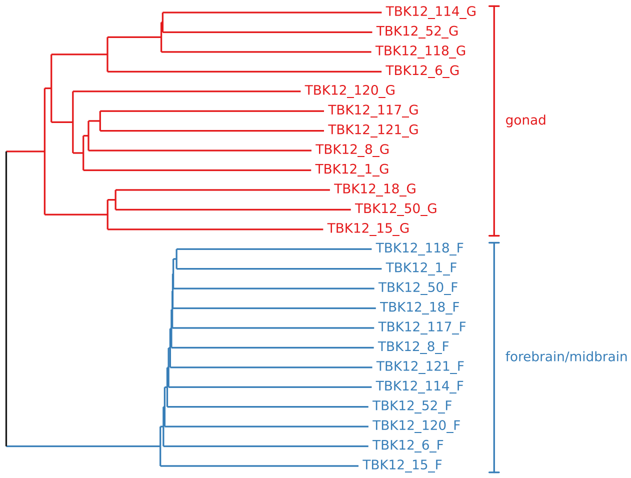

Fig. S4 Midpoint-rooted neighbor-joining tree obtained from the distance matrix computed by Cnidaria for the set of 24 bluehead wrasse samples. Clade colors denote tissue type.

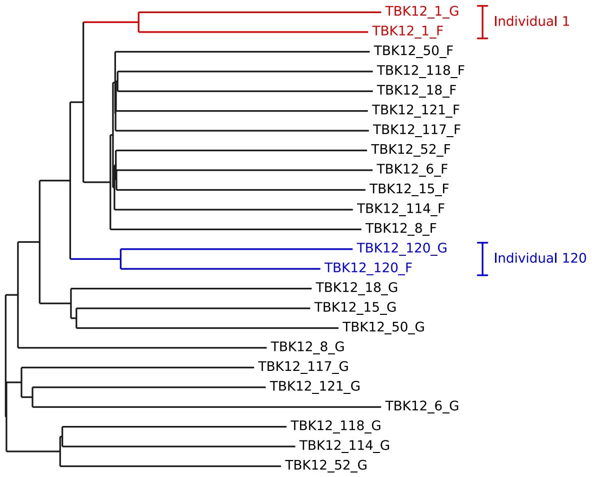

Fig. S5 Midpoint-rooted neighbor-joining tree obtained from the distance matrix computed by Cnidaria for the set of 24 bluehead wrasse samples, after filtering transcripts to include only genes in ideal components. Clade colors denote individual. Only clades which contain all samples for an individual and contain exclusively samples for that individual are color coded.
